# Kidney diseases and long non-coding RNAs in the limelight

**DOI:** 10.3389/fphys.2022.932693

**Published:** 2022-10-10

**Authors:** Chenxin Liu, Kuai Ma, Yunchao Zhang, Xing He, Linjiang Song, Mingxuan Chi, Zhongyu Han, Guanhua Li, Qinxiu Zhang, Chi Liu

**Affiliations:** ^1^ Reproductive and Women-Children Hospital, School of Medical and Life Sciences, Chengdu University of Traditional Chinese Medicine, Chengdu, China; ^2^ Department of Nephrology, Osaka University Graduate School of Medicine, Osaka, Japan; ^3^ School of Clinical Medicine, Chengdu Medical College, Chengdu, China; ^4^ Department of Nephrology, Sichuan Academy of Medical Science and Sichuan Provincial People’s Hospital, Sichuan Renal Disease Clinical Research Center, University of Electronic Science and Technology of China, Chengdu, China; ^5^ Chinese Academy of Sciences Sichuan Translational Medicine Research Hospital, Chengdu, China; ^6^ Department of Cardiovascular Surgery, Sun Yat-sen Memorial Hospital, Sun Yat-sen University, Guangzhou, Guangdong, China; ^7^ Hospital of Chengdu University of Traditional Chinese Medicine, Chengdu, China

**Keywords:** long non-coding RNA, chronic kidney disease, IgA nephropathy, biomaker, membranous nephropathy, acute kidney injury, lupus nephritis, diabetic nephropathy

## Abstract

The most extensively and well-investigated sequences in the human genome are protein-coding genes, while large numbers of non-coding sequences exist in the human body and are even more diverse with more potential roles than coding sequences. With the unveiling of non-coding RNA research, long-stranded non-coding RNAs (lncRNAs), a class of transcripts >200 nucleotides in length primarily expressed in the nucleus and rarely in the cytoplasm, have drawn our attention. LncRNAs are involved in various levels of gene regulatory processes, including but not limited to promoter activity, epigenetics, translation and transcription efficiency, and intracellular transport. They are also dysregulated in various pathophysiological processes, especially in diseases and cancers involving genomic imprinting. In recent years, numerous studies have linked lncRNAs to the pathophysiology of various kidney diseases. This review summarizes the molecular mechanisms involved in lncRNAs, their impact on kidney diseases, and associated complications, as well as the value of lncRNAs as emerging biomarkers for the prevention and prognosis of kidney diseases, suggesting their potential as new therapeutic tools.

## Introduction

Deaths from kidney disease are increasing at an alarming rate worldwide. Without timely intervention, both chronic and acute kidney disease can progress to end-stage renal disease (ESRD). With no practical, cost-effective treatments available, the current best response strategy for ESRD is to prevent, diagnose, and treat early to delay or prevent its progression. The clinical diagnosis of kidney diseases still greatly relies on glomerular filtration rate (GFR) and serum creatinine and urea levels, which may result in a missed diagnosis, especially in the early stages of onset, delaying diagnosis and treatment (Webster et al., 2017). Moreover, various classes of kidney-protective drugs are not uniformly effective (Muskiet et al., 2019). More potential novel tests and diagnostic and therapeutic approaches therefore need to be explored.

Long non-coding RNAs (lncRNAs) are untranslated regulatory RNAs that regulate cellular processes, including transcriptional interference and activation; nuclear transcription; and a variety of biological processes such as genomic and chromosomal modifications, epigenetics, cell cycle, and cell differentiation (Gong and Maquat, 2011; Kim et al., 2014; Böhmdorfer and Wierzbicki, 2015; Chen et al., 2021; Statello et al., 2021). Abundant studies have shown that lncRNAs express at different levels in kidney diseases and are involved in all stages of them. LncRNAs have been highlighted as diagnostic biomarkers, and targeting lncRNAs may represent a precise therapeutic strategy for progressive kidney diseases. LncRNAs may exist in a stable form in serum and urine as biomarkers. They show varying expression levels in different diseases and are associated with pathways, targets, and events involved in the pathophysiology of kidney disease, which is key to their use as biomarkers.

In this review, we provide a comprehensive overview of the current biogenesis, degradation, and functions of lncRNAs and highlight current knowledge about their functional roles in kidney diseases—including chronic kidney disease (CKD), membranous nephropathy (MN), immunoglobulin (Ig)A nephropathy (IgAN), lupus nephritis (LN), diabetic nephropathy (DN), and acute kidney injury (AKI)—and their complications and inheritance. We also mention some lncRNA-targeted therapeutic drugs and some lncRNA differential expression studies. Finally, we highlight some of the major mechanisms of lncRNAs in different kidney diseases and prospected future research directions.

## Biogenesis, degradation, and exosome formation of LncRNAs

LncRNAs are a class of untranslated regulatory RNAs >200 nucleotide sequences in length that are poorly explored but widely present in the human body. The NONCODEV5 datasets show that there are >100,000 lncRNAs in the human genome (Hon et al., 2017). LncRNAs are 70%–98% abundant in cells and generally specific to primates and cells, with lower levels of expression and less selective restrictions than protein-coding genes. (Derrien et al., 2012; Alessio et al., 2020). Previous studies have reported that lncRNAs are unable to encode proteins, but recent investigations have identified abundant small open reading frames in lncRNAs *via* ribosomal analysis, some of which have the potential to encode micro-peptides and proteins (Kong et al., 2020; Ye et al., 2020; Zhou B. et al., 2021; Kute et al., 2021).

RNA polymerase II (Pol II) transcribes protein-encoding genes and numerous pre–messenger RNAs (mRNAs) of lncRNAs, including long intergenic non-coding RNAs (lincRNAs), enhancer RNAs, antisense RNAs, and promoter upstream transcripts (Nojima and Proudfoot, 2022). LncRNA is mainly transcribed by RNA Pol II and was initially thought to be the byproduct and noise of Pol II transcription. Subsequent studies confirmed that lncRNA is involved in X chromosome silencing, genomic imprinting, chromosome modification, and transcriptional activation and interference (Brockdorff et al., 1992; Ntini et al., 2018). LncRNA has also been found to be transcribed by RNA polymerase III, and smaller lncRNAs can be processed post-transcriptionally from introns.

The Cap structure (m7GpppG) is added to the 5′ end of the Pol II transcription unit. However, the 3′ end of many lncRNA transcription units are only partially cleaved and polyadenylated by the polyadenylation complex at the functional polyadenylation site. Almost 98% of lncRNAs are spliced and sheared similarly to mRNAs, and some share similar structures with classical mRNAs (Derrien et al., 2012) (van Heesch et al., 2014). Most non-polyadenylated lncRNAs are rapidly degraded by the RNA exocytosis complex, which is the primary mechanism of their degradation and a mechanism of accumulation in chromatin (Schlackow et al., 2017). A minority of lncRNAs are transcribed by dysregulated Pol II, retained on chromatin, and degraded by exosomes (Statello et al., 2021). Some undegraded lncRNAs may be post-processed into smaller RNAs, especially small nucleolar RNAs (Derrien et al., 2012).

Statistically, lncRNAs are mostly enriched in the cytoplasm, especially in ribosomes. LncRNAs reaching the cytoplasm will come to the default destination ribosome and may be degraded therein (van Heesch et al., 2014; Carlevaro-Fita et al., 2016). LncRNAs participate in forming partial nucleolus networks, regulating ribosome genesis, and modulating the function and structure of the nucleolus. LncRNA facilitates regulatory crosstalk of RNA Pol II transcription with the nucleolus, and some lncRNAs are encoded on the human ribosomal DNA locus (Vydzhak et al., 2020; Mamontova et al., 2021).

Post-transcriptional lncRNAs undergo nuclear retention due to various mechanisms like transcriptional inefficiency, differential expression of specific splicing factors, and recruitment to nuclear factors. Some lncRNAs share processing and export pathways with mRNAs. LncRNA genes have fewer exons than mRNAs but surprisingly present only two exons at a ratio of 7 times that of protein-coding genes (Derrien et al., 2012). Transcripts with longer or fewer exons preferentially depend on nuclear RNA export factor 1 for nuclear RNA export, and lncRNA is commonly subject to reversible post-transcriptional modification of N6-methyladenosine, a region of modification that drives the export of single-exon transcripts (Zuckerman et al., 2020).

There are >1,500 genes encoding for RNA-binding proteins (RBPs) in the human genome, and various RBPs interact with lncRNAs transported to the cytoplasm and coordinate post-transcriptional gene regulation and RNA processing with ribonucleoproteins (Gerstberger et al., 2014). Some lncRNAs are exported from the nucleus, then sorted into the mitochondria by an unspecified mechanism. Recently, it was reported that lncRNA is involved in multiple pathways of mitochondrial autophagy, affecting some degenerative pathologies, cardiovascular disease (CVD), and cancer (Li Y. et al., 2021; Tai et al., 2022). Besides the nucleus-encoded lncRNA localized to mitochondria, some mitochondrial DNA-encoded lncRNAs were also identified (Liang et al., 2021).

**FIGURE 1:**
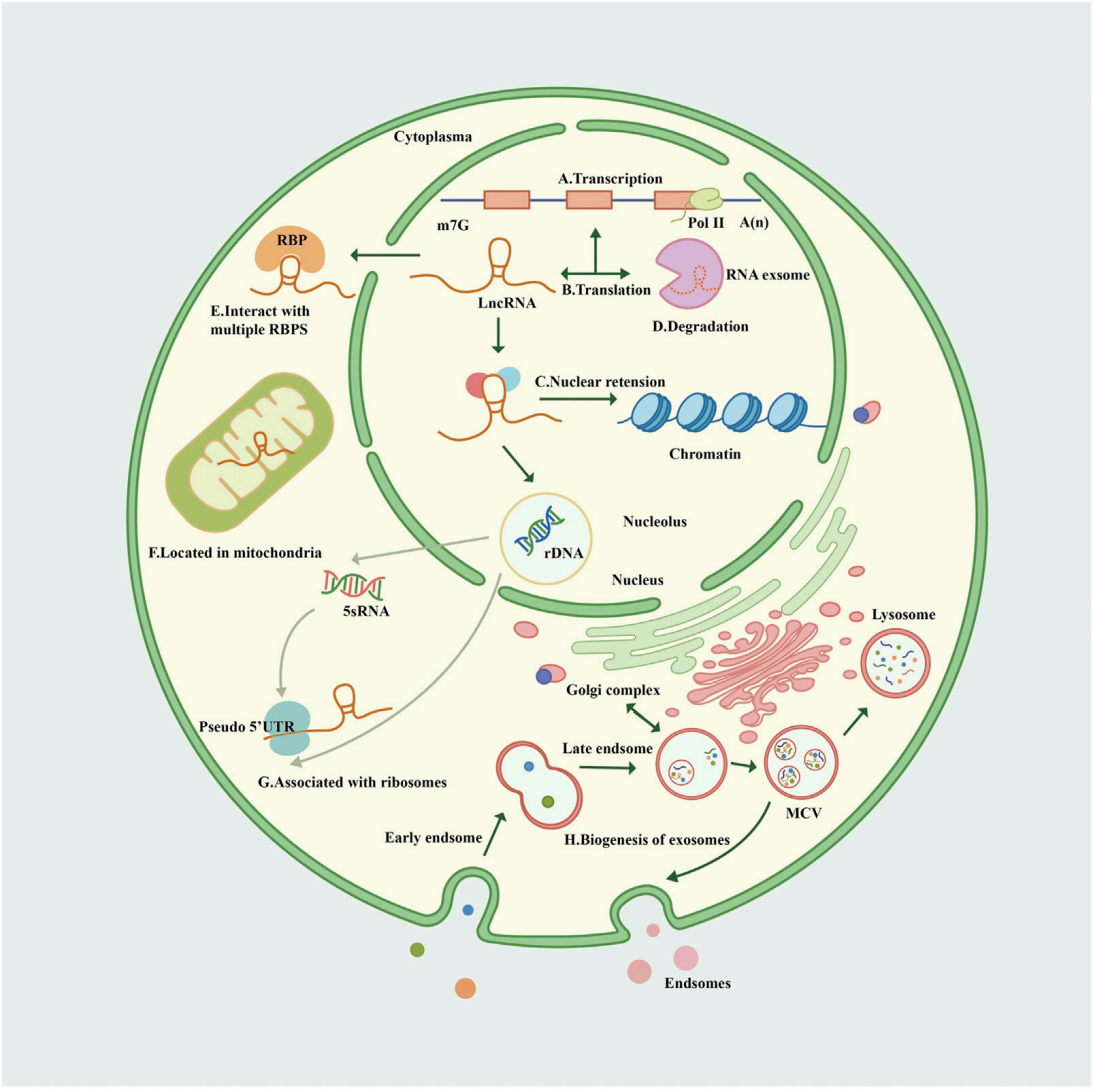
Biogenesis and cellular routes of lncRNAs. **(A)**. The transcription of lncRNA. LncRNA is mainly transcribed by Pol II with a Cap structure at the 5′ end and generally a non-characteristic poly A structure at the 3′ end. **(B)**. The translation of lncRNA. Some open reading frames on lncRNA translate some functional peptides and proteins. **(C)**. LncRNA has nuclear retention and nuclear localization through various mechanisms. **(D)**. The degradation of lncRNA. Most non-polyadenylated lncRNAs are rapidly degraded by the RNA extracellular complexes, and some lncRNAs are transcribed by dysregulated Pol II, retained on chromatin, and also degraded by exosomes. **(E)**. LncRNAs interact with multiple RBPS in the cytoplasm. **(F)**. Lncrnas are sorted into mitochondria through unknown mechanisms. **(G)**. Many lncRNAs in the cytoplasm are related to ribosomes through “pseudo” 5′ untranslated regions (UTRs); Lncrna participates in the process of nucleolus ribosomes, which may explain the fact that lncRNA is associated with ribosomes. **(H)**. Nanoscale membrane vesicles are released to form exosomes through the fusion of plasma membrane through the endocytosis pathway, and the mechanism of lncRNA sorting into exosomes remains unclear, but it may be related to Golgi apparatus. Exosomes can be degraded by lysosomes. Pol II, polymerase II; RBP, RNA-binding protein; UTR, untranslated region; MVB, multivesicular body.

LncRNA exosomes are detectable in human blood, urine, and other body fluids as biomarkers (Wang Y. et al., 2021; Yu et al., 2022). Exosomes are endosomal-derived extracellular vesicles and organelles (multivesicular bodies) that release nanoscale membrane vesicles by fusion of the plasma membrane *via* the endocytic pathway (Hessvik and Llorente, 2018). The mechanism of lncRNA sorting into exosomes is unclear; the Golgi apparatus, lysosomes, and several RBPs (including the heterogeneous nuclear ribonucleoprotein family and human antigen R) have been demonstrated to be involved in the sorting process of lncRNA (Yuan and Huang, 2021). In a study of competing endogenous RNA (ceRNA) networks, lncRNA exosomes were linked to the extracellular matrix (ECM), immune system processes, catabolic processes, and signal transduction in the kidneys (Zhou S. et al., 2021) (Figure 1).

## Functions and mechanisms of LncRNAs

Nowadays, many more functions are surfacing as lncRNAs are increasingly further explored. LncRNA function is often mediated by modular structural domains, including sequence motifs and secondary structures that interact with other RNAs, DNA, or proteins. LncRNAs can be classified as sense overlap, exon–intron antisense, bidirectional, intergenic, natural antisense, or intron–sense overlap based on their position relative to protein-coding genes (Kirk et al., 2018; Kopp and Mendell, 2018).

LncRNA generally functions at epigenetic, transcriptional regulation, and post-transcriptional regulation levels. Specific lncRNAs recruit chromosomal remodeling and modification complexes at certain sites (Khalil et al., 2009; Statello et al., 2021). LncRNAs can also be regulated at the transcriptional level through a variety of mechanisms; for example, lncRNA transcription can interfere with the expression of neighboring genes, act as a co-factor to regulate the activity of transcription factors, and even produce enhancer RNAs, which can regulate in specific directions or over long distances (Statello et al., 2021). In parallel to these two mechanisms, lncRNAs can also function by participating in mRNA post-transcriptional regulatory processes, such as variable shear, RNA editing, protein translation, and translocation (Dykes and Emanueli, 2017).

LncRNAs regulate the expression of target genes through decoy, signaling, guidance, and scaffold mechanisms. LncRNAs competitively adsorb and bind large amounts of RNAs (e.g., microRNA [miRNA]), thereby releasing specific DNA regions or target proteins to other interactors, buffering and reducing their ability to interfere with mRNA-encoded proteins of target genes, and rendering lncRNA and mRNA interrelated as ceRNAs (Yao et al., 2019; Cervena et al., 2022). LncRNAs respond to intracellular and extracellular signaling pathways and act as regulators of signaling pathways, enabling convergence and integration of information among different signaling pathways at varying points of time and space (Yang et al., 2014; Statello et al., 2021). LncRNA-mediated transcriptional regulation can affect transcription, mRNA stability, or translation through cis- or transactivation patterns (Yao et al., 2019).

A growing number of lncRNAs have shown the ability to encode proteins, which may relate to their mechanisms in diseases. Due to the differential and specific expressions of lncRNAs in different species, life stages, tissues, and organs, we speculate that lncRNAs have better potential as biomarkers and may play a role in different cells, tissues, and life cycles (Derrien et al., 2012).

## LncRNAs and CKD

CKD is a common disease with high prevalence, morbidity, and mortality rates worldwide (2020; George et al., 2022). Patients with CKD gradually lose kidney function and eventually develop ESRD, also known as uremia. During *in vivo* experiments, unilateral ureteral obstruction (UUO) mimics human chronic obstructive nephropathy in an accelerated manner and is often used as a classic model of mouse renal fibrosis and CKD, which is characterized in terms of phenotypic symptoms by hypoperfusion-induced tubular ischemia, interstitial fibrosis, and altered renal function ( Washino et al., 2020; Jiao et al., 2021 ).

Renal fibrosis is a devastating manifestation of CKD, and lncRNA is associated with it *via* the anti-fibrotic growth factor transforming growth factor–β (TGF-β), which promotes renal fibrosis and can act on CKD kidneys through both classical and non-classical pathways, with Smads TGF-β classical signaling (mainly TGF-β1) holding a central role in the development of renal fibrosis (Jones et al., 2019). The Smad proteins (Smad2, Smad3, and Smad4) play different or even opposing functions in the regulation of fibrosis (Meng et al., 2016). Smad3 exerts a pro-fibrotic function and induces transcription of pro-fibrotic miRNAs and lncRNAs (Gu et al., 2020a). LncRNA can regulate the expression of some fibrosis-related genes and proteins, such as collagen, α–smooth muscle actin, and fibronectin. One study showed that 625 lncRNAs were up-regulated in the urine of UUO rats, and 19 up-regulated lncRNAs were predicted to contain several conserved Smad3-binding motifs in their promoters. Among them, TGF-β significantly induced promoters of lncRNAs containing >4 conserved Smad3-binding motifs in normal rat renal tubular epithelial NRK-52E cells (Zhou et al., 2018). TGF-β/Smad3-interacting lncRNA blocks the interaction of Smad3 with TGF-β receptor I by binding to the MH2 domain of Smad3. This indicates that lncRNA can specifically inhibit TGF-β–induced Smad3 phosphorylation and downstream pro-fibrotic gene expression from alleviating renal fibrosis (Wang et al., 2018). LncRNA-regulated genes may also contribute to the anti-renal fibrotic effect under norcantharidin treatment (Xiao H. et al., 2019). Confirmation exists that lncRNAs TCONS_00088786 and TCONS_01496394, which are regulated by TGF-β stimulation, can affect the expression of some fibrosis-related genes through a feedback loop. After TCONS_00088786 knockdown, concentrations of miR-132 and fibrosis-related proteins collagen I and III were reduced (Zhou et al., 2018). Similarly, a novel lncRNA, Erbb4-immunoreactivity (Erbb4-IR), is induced by TGF-β1 through a Smad3-dependent mechanism and highly up-regulated in fibrotic kidneys in UUO mice; therefore, silencing Erbb4-IR blocked TGF-β1–induced collagen I and α–smooth muscle actin expression *in vitro* (Feng et al., 2018). In addition, Erbb4-IR can also target Smad7, and specific silencing of Erbb4-IR expression up-regulates Smad7 in the kidneys, thereby attenuating TGF-β1/Smad3-induced renal fibrosis *in vivo* and *in vitro* (Gu et al., 2020a; Xia W. et al., 2021). Some lncRNAs, such as H19 and lncRNA plasmacytoma variant translocation 1 (PVT1), target miRNAs and act on downstream pathways to exert their effects. Of these, the former, together with miR-17 and fibronectin, constitutes a regulatory network involved in renal fibrosis (Xie et al., 2016), while the latter is mainly regulated by the miR-181a-5p/TGF-βR1 signaling pathway and knock out PVT1 can inhibit renal fibrosis (Cao L. et al., 2020).

Oxidative damage and inflammatory damage are also involved in the process of renal fibrosis. LncRNAs can affect the expression of some inflammasomes and are associated with reactive oxygen species (ROS) production and defense. The lncRNA X inactive specific transcript (XIST) mitigates renal inflammation, and ROS production induces oxidative damage in calcium oxalate–induced renal calcinosis by targeting miR-223 and NOD-like receptor protein 3 (NLRP3) (Lv et al., 2021). The lncRNA segment H19 functional molecule is involved in the regulation of the high motor group box 1/Toll-like receptor 4 (TLR4)/nuclear factor kappa B (NF-ĸB) cell signaling pathway, and the expression of the lncRNA H19 was significantly correlated with oxidative stress, mineralization (phosphorus, parathyroid hormone, organic carbon), and inflammatory markers such as tumor necrosis factor-α (TNF-α) and interleukin (IL)-6 in CKD patients (Fan et al., 2019; Okuyan et al., 2021). Yang et al. isolated the natural phenolic acid compound protocatechualdehyde from *Salvia miltiorrhiza* and found that it inhibited the expression of the Smad3-dependent lncRNA 9884; inhibited the abnormal expression of inflammatory cytokines iNOS, McP-1, and TNF-α in renal tubular epithelial cells; and attenuated UUO (Yang et al., 2021). Other studies have also identified many novel molecular targets for TGF-β1–mediated nephritides, such as lncRNA np_4334 (also known as Ptprd-IR). Ptprd-IR was also found to stimulate inflammatory responses in the kidneys, and its knockdown in renal tubular epithelial cell cultures blocked TGF-β1 and IL-1β–mediated NF-κB inflammatory signaling but did not affect TGF-β1–triggered Smad3 pathway activity and fibrosis (Pu et al., 2020).

A series of biological processes, such as cell proliferation, apoptosis, autophagy, and epithelial–mesenchymal transition (EMT), can drive renal fibrosis, which can be regulated by lncRNAs. The lncRNA LINC00667 reduces proliferation and invasion of CKD cells, increasing the apoptosis rate by regulating miR-34c. Reducing LINC00667 also promotes renal tubular epithelial cell proliferation and ameliorates renal fibrosis *via* the miR-19b-3p/LINC00667/connective tissue growth factor signaling pathway (Chen W. et al., 2019; Huang P. et al., 2021). In an animal experiment, SRY–Box transcription factor 6 (SOX6) was up-regulated in UUO mouse kidneys. A dual-luciferase reporter gene assay showed that XIST directly binds to miR-19b, then targets and up-regulates SOX6, forming the lncRNA XIST/miR-19b/SOX6 signaling axis. Therefore, XIST knockdown can up-regulate miR-19b, thereby inhibiting the expression of SOX6, which in turn improves apoptosis and fibrosis in UUO mouse kidneys (Xia W. P. et al., 2021). The lncRNA 74.1 was significantly down-regulated in clinical renal fibrosis specimens and promoted ROS defense by activating prosurvival autophagy, then decreased the ECM-related proteins fibronectin and collagen I involved in renal fibrosis (Xiao X. et al., 2019). The lncRNA myocardial infarction–associated transcript (MIAT) is up-regulated in UUO model mice and promotes cell viability, proliferation, migration, and EMT by regulating the miR-145/EIF5A2 axis (Wang et al., 2020c). Studies have also shown that silencing of MIAT attenuates myofibroblast formation. The regulatory role of lncRNAs in myofibroblast formation and their interaction with miRNA can improve renal fibrosis, which provides a therapeutic idea for UUO-induced renal–interstitial fibrosis (RIF) (Bijkerk et al., 2019). The lncRNA homeobox transcript antisense RNA (HOTAIR) was also up-regulated in UUO rats, and silencing it could up-regulate miR-124 and block the Notch1 pathway, thereby attenuating EMT and RIF, suggesting that HOTAIR can be used as a therapeutic agent for RIF (Zhou H. et al., 2019).

LncRNAs are also involved in another important mechanism of pathological injury in CKD–cell pyroptosis, which is a mode of programmed cell death that encompasses some of the characteristics of apoptosis and necrosis. Pyroptosis can affect CKD development through both classical and non-classical pathways; the classic pathway is cysteine-containing aspartate-specific protease–1 (caspase-1)–dependent cell death, which contains multiple inflammasome pathways, and the non-classical pathway mainly relies on the cell death pattern of caspase-4/5/11 (Shi et al., 2015; Mu et al., 2022). The caspase family are mainly associated with the release of inflammatory factors in CKD and pyroptosis of renal tubular epithelial cells. LncRNAs such as metastasis-associated lung adenocarcinoma transcript 1 (MALAT1) promote pyroptosis by down-regulating miR-23c and targeting its downstream gene cell scorch death-associated protein ELAV-like protein 1. In combination, MALAT1 negatively regulates caspase-1 and affects its classical pyroptosis pathway. Another lncRNA, growth arrest-specific 5 (GAS5), has anti-pyroptotic properties, while the target molecule of GAS5, miR-452-5p, has pro-pyroptotic properties (Ding et al., 2021).

The involvement of lncRNAs has also been confirmed in kidney physiogenesis. The onset of CKD is closely associated with poor growth and development early in life. The kidneys are very sensitive to prenatal injury, and various maternal exposure factors lead to kidney reduction and fewer kidney units, which are strongly associated with increased blood pressure and CKD development (Briffa et al., 2020). During rat spermatogenesis, a high-fat, -sucrose, and -salt diet resulted in reduced GFRs in both sexes of the filial generation 2 (F2) generation and CKD in female offspring, during which differential lncRNA expression occurred. Expression of the lncRNA XR-146683.1 was decreased in female F0HD+F1HD (high-fat diet) F2 offspring and correlated with *Tmem144* gene expression. This suggests that lncRNA may be involved in the process of epigenetic alteration of renal gene expression due to an unhealthy diet, but its relevance and specific mechanisms require further investigation (Zhang et al., 2022).

**FIGURE 2:**
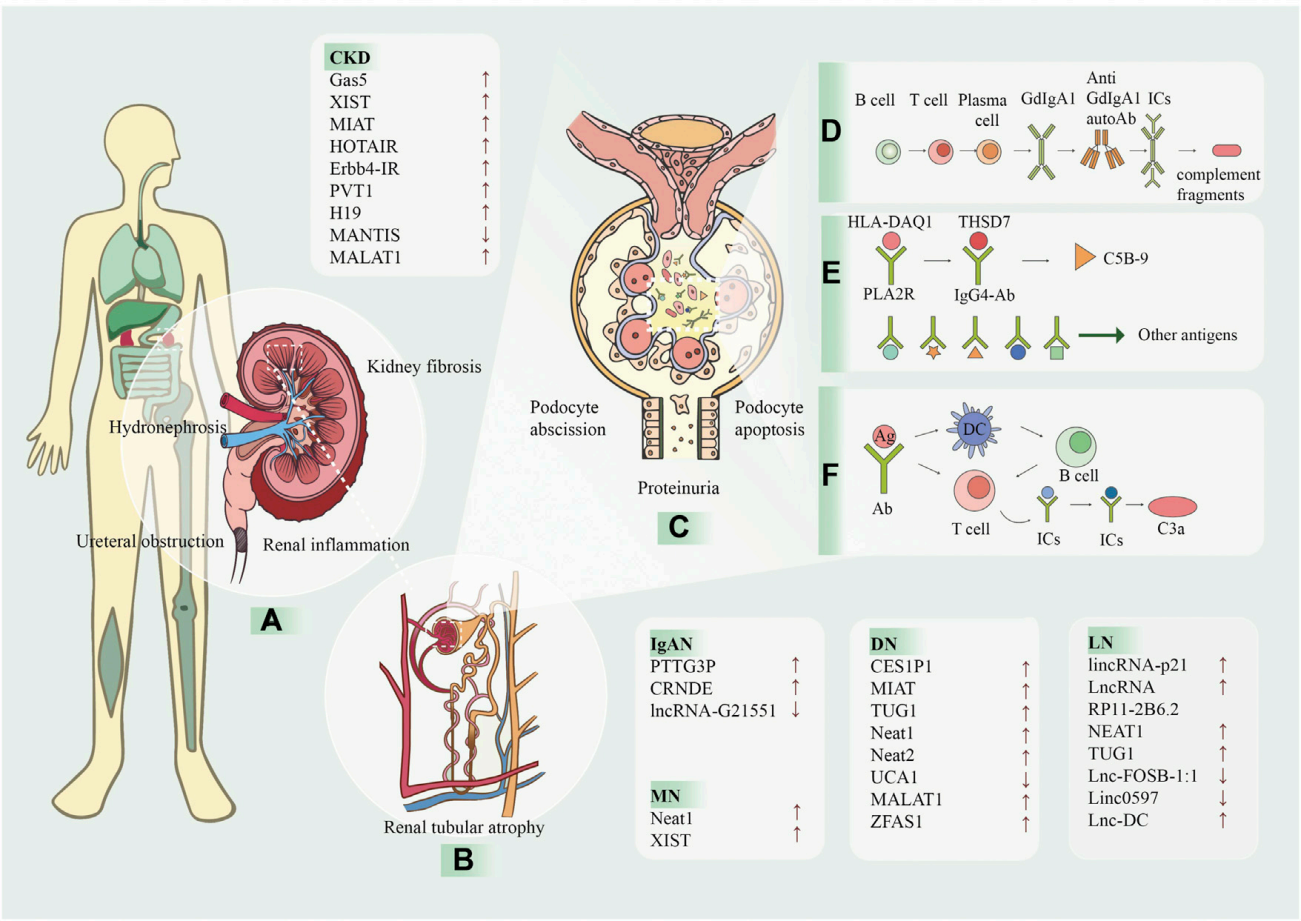
The processes of kidney diseases and the involvement of lncRNA. LncRNA has been proven to be associated with a variety of kidney diseases and can be used as a biomarker for prevention and detection. The arrows after the text indicate the lncRNA levels increase (↑) or decrease (↓). **(A)** CKD is caused by many reasons, and one of the main pathological manifestations is renal fibrosis and renal tubular atrophy, which causes the kidney to be dark and bumpy. **(B)** Kidney diseases are caused by many reasons, and one of the main pathological manifestations is renal fibrosis and renal tubular atrophy. **(C)** The following ICs and complement are deposited in the mesangial region and are attacked by the autoimmune system, causing kidney damage. In addition, apoptosis and shedding of podocytes, renal tubular epithelial cells, partial compensatory hyperplasia and proteinuria occur. **(D)** In IgAN, T and B cells act on plasma cells to produce glycosylated IgA1, which forms ICs with antibodies and is deposited in mesangial region. **(E)** In MN, HLA-DAQ1 binds to PLA2R as an antigen, THSD7A binds to IgG4-AB, and activates the C5B-9 fragment of complement. In addition, some small molecules, such as bovine serum albumin, anti-neutral endopeptidase and superoxide dismutase two can also be used as antigens. **(F)** In LN, antigen is presented to T cells by DC, and can also interact directly with B cells to produce antigen-antibody complexes and generate C3a complement fragments. CKD, chronic kidney disease; MN, membranous nephropathy; IgAN, immunoglobulin A nephropathy; LN, Lupus nephritis; Gd-lgA1, galactose-deficient IgA1; ICs, immune complexes; HLA, human leukocyte antigen; PLA2R, podocyte receptor and M-type phospholipase A2 receptor; THSD7A, thrombin reactive protein type 1 domain-containing 7A; IgG4, immunoglobulin G 4; Ag, antigen; Ab, antibody; DC, dendritic cell.

Many other lncRNAs are differentially expressed in the CKD and UUO models and may serve as potential biomarkers. In a study using transcriptome sequencing data, bioinformatics analysis, and qRT-PCR assays, 24 lncRNAs were up-regulated and 79 lncRNAs were down-regulated in UUO rat kidney tissues (Sun et al., 2017) (Figure 2).

In recent years, secondary nephropathy caused by diabetes mellitus (DM) and hypertension has become a primary cause of CKD. The role of lncRNAs in DN and hypertensive nephropathy has been investigated extensively (Guo et al., 2019; Wang et al., 2020b; Ren and Wang, 2021). LncRNA SNHG12, taurine up-regulated gene 1 (TUG1) and AK094457 participate in angiotensin II induced hypertensive vascular endothelial injury and renal fibrosis ( Xu et al., 2020; Qian et al., 2021; Zhang et al., 2021 ). LncRNAs play a major role in epigenetics, includeing cancer, cardiovascular disease and are involved in the diabetes-related CKD process by regulating epigenetic enzymes such as DNA methyltransferases, histone deacetylases, and histone methyltransferases (Li N. et al., 2022; Shelke et al., 2022). Epigenetic regulation, including ncRNAs, histone modifications, an, is closely associated with CKD caused by secondary disease, but the specific mechanisms are unclear (Lu et al., 2017; Kato and Natarajan, 2019). The emerging role of lncRNAs in the epigenetics and epigenomics of CKD and the potential for detection and treatment deserve in-depth exploration.

## LncRNA in IgAN

IgAN is a disease in which immune complexes (ICs) mainly formed by IgA are deposited in the mesangial area of the glomerulus, causing glomerular inflammation and additional kidney damage (Tortajada et al., 2017). It is considered the most common form of primary glomerulonephritis (Chen L. et al., 2019). Accumulating evidence suggests that lncRNAs play a role in diverse cellular functions, including cell growth, invasion, migration, metabolism, apoptosis, and differentiation (Li et al., 2017).

In IgAN, lncRNAs are not only involved in the regulation of cytokines and inflammation but also related to B-cells and macrophages. The lncRNA PTTG3P induces abnormal production of glycosylated IgA1; moreover, PTTG3P was overexpressed in IgAN samples compared to healthy subjects, and urinary PTTG3P levels were also higher in IgAN patients than healthy controls. Meanwhile, PTTG3P overexpression stimulated B-cell growth, enhanced the expressions of cyclin miRNA and Ki-67, and induced the production of IL-1β and IL-8 (Bi et al., 2021). Previous studies have shown that IL-1β and IL8 play key roles in the occurrence and development of IgAN; thus, PTTG3P plays a key role in the progression of IgAN (An et al., 2020). LncRNA colorectal neoplasia differentially expressed (CRNDE) exacerbates IgAN progression by inhibiting nucleotide-binding oligomerization NLRP3 ubiquitination and degradation, promoting NLRP3 inflammasome activation in macrophages (Shen et al., 2021).

LncRNAs are potential novel non-invasive IgAN biomarkers. To date, renal biopsy remains the standard for the diagnosis of IgAN, without effective disease-specific treatments. However, renal biopsy carries many limitations in assessing disease activity at the time of biopsy alone (Moresco et al., 2015). Therefore, finding non-invasive biomarkers is urgent for the diagnosis of IgAN. A study indicated that a total of 149 lncRNAs interact with seven mRNAs associated with the “innate immune response” Gene Ontology term, demonstrating that differentially expressed lncRNAs and mRNAs may have a role in IgAN development. The expression of the lncRNA MYEF2-1.1 was 85-fold lower in IgAN patients than healthy controls, while that of ALOX15P1-ncNR045985 was 5.15-fold higher (Zuo et al., 2017). Other researchers identified the lncRNA G21551 as a potential diagnostic biomarker for IgAN by high-throughput RNA sequencing (Guo et al., 2020).

The co-expression networks generated in some studies suggest the mutual regulation of lncRNAs and mRNAs in IgAN patients and that lncRNAs and mRNAs co-regulate IgAN (Zuo et al., 2017). The mutual regulation of lncRNAs and mRNAs may also be involved in IgAN-related innate immune disorders. Dysregulation in the lncRNA–mRNA network may be a potential mechanism of IgAN progression (Wang et al., 2013). These findings lay the foundation for the study of lncRNAs in kidney disease. The functions and regulatory mechanisms of lncRNAs in IgAN should be further explored to identify potential screening biomarkers and new therapeutic targets.

## LncRNA in MN

MN is a kidney-specific autoimmune disease (Couser, 2017). Podocyte receptor and M-type phospholipase A2 receptor and thrombin reactive protein type 1 domain-containing 7A have been identified as auto-antigens related to glomerular deposition mainly bound by IgG4 anti-autoantibodies (Meyer-Schwesinger et al., 2015; Couser, 2017). The formation of antigen–antibody complexes leads to glomerular damage, podocyte apoptosis, and autophagy, activating the complement system and resulting in increased basement membrane permeability and massive proteinuria (Couser et al., 1985; Li Y. et al., 2022; Gao et al., 2022; Hoxha et al., 2022).

LncRNA can affect the MN process by regulating cell apoptosis. In experimental MN mouse models, lncRNA dysregulation, such as NEAT1 and XIST, has been identified. NEAT1 was time-dependently up-regulated in albumin-stimulated MN tubular tissue. However, NEAT1 can inhibit Noxa (a Bcl-2 homolog 3–only protein)-mediated anti-apoptotic protein activity to induce apoptosis and promote MN development (Pi et al., 2021). XIST is up-regulated in the renal tissue of MN patients and up-regulates TLR4 by negatively regulating miR-217. Up-regulation of TLR4 expression promotes podocyte apoptosis and MN development. Conversely, down-regulation of XIST can improve podocyte apoptosis through the miR-217/TLR4 pathway, which may improve MN (Jin L. W. et al., 2019). The reduction of histone H3 trimethylation at lysine 27 (H3K27me3) in the XIST promoter region leads to increased urinary XIST levels, which could be a biomarker for MN detection (Huang et al., 2014).

LncRNAs are involved in the construction of lncRNA–miRNA–mRNA co-expression networks and then ceRNA networks. For example, LINC00052 is involved in three ceRNA pathways in the ceRNA network, i.e., LINC00052–hsa-miR-145-5p–EPB41L5, LINC00052–hsa-miR-148a-3p–FAM43A, and LINC00641–hsa-497-5p–PRKG1. The deletion of EPB41L5 causes renal failure and nephrotic syndrome. Hsa-miR-145-5p and miR-148a-3p are dysregulated in renal cell carcinoma, while miR-497-5p can inhibit cell proliferation and promote apoptosis in renal clear cell carcinoma. Meanwhile, LINC00641 can regulate PRKG1 by sponging hsa-497-5p as a ceRNA (Zhou G. et al., 2021). Therefore, dysregulated ceRNAs formed by lncRNAs may be involved in MN development. Additionally, miRNAs associated with MN-related immune genes and lncRNAs were predicted in a study of differentially expressed mRNAs and MN-related immune genes in MN. Moreover, the interaction of ceRNA was explored through lncRNA–miRNA–mRNA network construction. In the KCNQ1OT1–miR-204-5p-SRY–Box transcription factor 4 (SOX4) pathway, mRNA SOX4 may promote MN progression through the interaction of sponge KCNQ1OT1–miR-204-5p (Li P. et al., 2021).

## LncRNA and LN

LN is a common complication of the chronic autoimmune disease systemic lupus erythematosus (SLE) with multiple pathogenic pathways, including abnormal apoptosis, auto-antibody production, and IC deposition with complement activation in the glomeruli, leading to inflammation and damage to the glomeruli (Chen et al., 2020). LncRNAs are associated with apoptosis, inflammation, and immune-related pathways in LN.

Dysregulation of lncRNAs accelerating apoptosis is a key pathogenesis of LN. During LN development, apoptosis is promoted by circulating lymphocytes, renal cells (renal tubules and glomerular parenchymal cells), and phagocytes used to remove apoptotic cells. In LN patients, the expression of lincRNA-P21 is up-regulated in peripheral blood monocytes and urine, while that of miR-181A in urine is down-regulated. The up-regulated expression of lincRNA-P21 leads to the death of apoptotic cells in circulating lymphocytes and renal cells, followed by the generation of auto-antibodies, leading to IC accumulation in SLE and glomerulonephritis formation. In addition, the renal expression of lincRNA-P21 increases gradually during LN development. *In vitro* experiments using CRISPR interference–lincRNA-P21 transfected cells showed a low rate of apoptotic cells in the presence of DNA damage response, suggesting a therapeutic strategy to treat LN by knocking down lincRNA-P21 expression to reduce apoptosis (Chen et al., 2020).

LncRNAs are involved in immune and inflammatory regulation in LN. LncRNA RP11-2B6.2 was found to be increased in the renal tissue of LN patients and positively correlated with disease activity and interferon (IFN) score. LncRNA RP11-2B6.2 leads to overactivation of IFN-I signaling in renal cells by epigenetic inhibition of SOCS1 (a negative regulator of IFN-I signaling) expression (Liao et al., 2019). Among the many pathogenic signaling pathways identified in LN, IFN-I response overactivation is closely associated with disease progression and prognosis (Psarras et al., 2017). Many molecules able to block the IFN-I signaling pathway have been developed to improve SLE symptoms, such as JAK inhibitors (tofacitinib) and monoclonal antibodies targeting IFN-α and IFN-I receptors. The lncRNA NEAT1 accelerates renal mesangial cell damage by regulating the miR-146b/TRAF6/NF-κB axis in LN. NEAT1 has a binding site for miR-146b. By directly sponging miR-146b to enhance the expression of TRAF6 in LN, TRAF6 activates the NF-κB signaling pathway in LN, which is a classic signaling pathway regulating immunity and inflammation, exacerbates the inflammatory damage of human renal mesangial cells, reduces their apoptosis, and accelerates renal injury (Zhang L. H. et al., 2020).

Some other lncRNAs are differentially expressed in LN patients and can serve as potential means to detect and predict the severity of disease. In a study of lncRNA expression profiles in neutrophils in SLE patients, 360 lncRNAs were up-regulated and 224 lncRNAs were down-regulated compared to in healthy controls. The lncRNA LNC-FSB-1:1 was further down-regulated in patients with LN. After 2 years of follow-up, lower LNC-FSB-1:1 expression was associated with a higher risk of LN within 2 years, suggesting that could be a potential biomarker for predicting recent kidney involvement (Cai et al., 2021). Linc0597 was up-regulated in patients with LN and positively correlated with the disease activity index, suggesting that it could also be used as an indicator of LN disease activity and diagnosis (Zheng et al., 2020). TUG1 is significantly reduced in SLE patients, especially those with LN, and can be used as a clinical diagnostic tool for SLE patients or SLE patients with LN (Liao et al., 2019). The protective effect of NF-κB inhibition on renal injury in mice with SLE may be related to lncRNA TUG1 and the inhibition of apoptosis and release of inflammatory factors, thus protecting HK-2 cells from lipopolysaccharide (LPS)-induced inflammatory injury (Cao H. Y. et al., 2020). Inc-DC is also differentially expressed in patients with LN and SLE, where its level appeared significantly higher than that in SLE patients without nephritis, and both groups’ levels were higher than that in healthy controls (Wu et al., 2017).

## LncRNA and DN

The deterioration of renal function caused by DM is known as DN, which is the most common cause of ESRD and a serious microvascular disease caused by DM. A high glucose environment promotes pathological changes of diabetic vascular complications by inducing oxidative stress, inflammation, metabolic abnormalities, and proliferation and angiogenesis in endothelial cells (ECs) (Wang Y. N. et al., 2021).

According to a systematic review and *in silico* analyses, five lncRNAs (MALAT1, GAS5, MIAT, CASC2, and NEAT1) participate in disease-related signal pathways, indicating their role in DN (Zhao et al., 2022). These lncRNAs are mainly involved in oxidative stress, inflammatory response, apoptosis, cell proliferation, pyroptosis, podocyte injury, and mitochondrial injury in DN (Gu et al., 2020b; Chu et al., 2022; Jia et al., 2022). We have summarized some lncNRAs related to DN (Table 1). The NLRP3 inflammasome is involved in the inflammatory response and closely related to DM and related complications. Studies have found that the up- or down-regulation of lncRNAs, such as lincRNA-GM4419, can inhibit the activation of the NLRP3 inflammasome and reduce the inflammatory response and is also associated with specific processes mediated by it, such as podocyte loss, glomerulosclerosis, and tubulointerstitial fibrosis, thereby improving DN (Lu X. et al., 2021). On the other hand, lncRNA is associated with DNA methylation, histone methylation, and acetylation in diabetic vascular complications (Lu J. et al., 2021).

**TABLE 1:** Levels, effects and functions of lncRNAs in experiments in kidney diseases.

Disease	LncRNA	Sample sources	Levels	Mechanisms	Effects	References
CKD	Erbb4-IR	kidney tissues of mouse	↑	down-regulates its target gene smad7	regulates TGF-β1-induced collagen I, alpha-smooth muscle actin (α-SMA) expressions and responsible for TGF-β/Smad3-mediated renal fibrosis	Feng et al. (2018)
	H19	kidney tissues and HK-2 cells of mouse	↑	regulates HMGB1/TLR4/NF-ĸB signaling pathway and forms a regulatory network with miR-17	regulates oxidative stress, mineralization, inflammation, and the progression of renal fibrosis in CKD	Xie et al. (2016), Fan et al. (2019), Okuyan et al. (2021)
	LINC00667	kidney tissues of patients with chronic renal failure	↑	acted upon by upstream miR-19b-3p and target connective tissue growth factor	down-regulates it can promote the proliferation, migration of tubule epithelial cells and participate in renal fibrosis	Chen et al. (2019a), Chen et al. (2019b)
		kidney tissues of patients with chronic kidney disease and HK-2 cells	↑	acted upon by upstream miR-34c	reduce the proliferation and invasion of chronic kidney disease cells, increase the apoptosis rate and improve renal fibrosis	Huang et al. (2021a), Huang et al. (2021b)
	LINC00963	kidney tissues of Wistar male rats	↑	targets gene forkhead box O	down-regulates it can suppress renal interstitial fibrosis (RIF) and oxidative stress (OS)	Chen et al. (2018)
	Atrolnc-1	muscles of mouse with cachexia and CKD	↑	alters NF-κB activity and MURF-1 expression NF-κB, and inhibits by insulin/IGF-1pathway	regulates muscle mass and proteolysis in CKD and initiate muscle wasting	Sun et al. (2018)
	MANTIS	serum, human umbilical vein endothelial cells (HUVECs) of patients with CKD	↓	inhibits the activation of p38 MAPK and p65 NF-κB pathways, positively regulates key endothelial gene SOX18	relieves the protein-bound uremic toxin-induced HUVECs injury in CKD and ESRD	Jiang et al. (2018)
	MALAT1	streptozotocin-induced diabetic rats and high-glucose-treated HK-2 cells	↑	down-regulates miR-23c and targets its downstream gene ELAVL1	negatively regulates caspase-1 and affects its classical pyroptosis pathway	Li et al. (2017), Ding et al. (2021)
	DKFZP43410714	plasma of patients with CKD at different stages	↑	inhibits the expression of intracellular adhesion molecule 1 (ICAM-1) and vascular cells adhesion molecule 1 (VCAM-1), increases the expression of endothelial nitric oxide synthase (eNOS)	reduces hypoxia-mediated endothelial cell apoptosis and monocyte adhesion, involved in the pathogenesis of endothelial dysfunction	Lai et al. (2019)
	Gas5	kidney tissues of UUO mouse models	↑	acts on PTEN/MMP-9 signaling pathway	deteriorates renal function and aggravates renal fibrosis	Guo et al. (2021)
	XIST	TGF-β1 treated HK-2 cells and kidney tissues of UUO mouse models	↑	acts on lncRNA XIST/miR-19b/SOX6 signal axis	regulates apoptosis and inflammation of renal fibrosis	Xia et al. (2021a), Xia et al. (2021b)
	Lnc MIAT	renal fibrotic tissues	↑	targets the miR-145/EIF5A2 axis	promotes cell viability, proliferation, migration, and the EMT, promotes RIF	Wang et al. (2020b)
	HOTAIR	kidney tissues of UUO mouse models	↑	regulates Notch1 pathway via the modulation of miR-124	promotes RIF	Zhou et al. (2019)
	LncRNA74.1	renal fibrosis specimens	↓	activates pro-survival autophagy then decreases ECM-related proteins, fibronectin and collagen I involved in renal fibrosis	promotes reactive oxygen species defense and alleviates renal fibrosis	Xiao et al. (2019)
	PVT1	renal fibrosis specimens	↑	via mediation of miR-181a-5p/TGF-βR1 axis	inhibits renal fibrogenesis	Cao et al. (2020b)
	lnc-TSI	kidney tissues of UUO mouse models	↓	negatively regulates the TGF-β/Smad3 pathway	inhibits renal fibrogenesis	Wang et al. (2018)
	Arid2-IR	kidney tissues of UUO mouse models	↑	targets NLR family CARD domain containing 5 (NLRC5) transcription	participates in interleukin-1β (IL-1β)-induced NF-κB activation and renal inflammation in vitro.	Zhou et al. (2015), Zhang et al. (2021)
	TCONS_00088786	kidney tissues of UUO mouse models	↑	targets miR-132 and reduces fibrosis-related protein	promotes renal fibrogenesis	Zhou et al. (2018)
IgAN	PTTG3P	IgAN samples and urinary	↑	promotes IL-1, IL-8 production via regulating miR-383, enhances cyclin D1 and ki-67 expression	induces aberrant glycosylated IgA1 production and B cell growth in IgA nephropathy	Bi et al. (2021)
	CRNDE	serum of IgAN patients, cell supernatants of in vitro IgAN model	↑	restrains ubiquitination and degradation of NLRP3 and facilitating NLRP3 inflammasome activation in macrophages	exacerbates IgA nephropathy	Shen et al. (2021)
	HOTAIR	a multilayer regulatory network	unknown	may lead to activation of NF-κB, resulting in an inflammatory response	acts as a potential non-invasive biomarker for IgAN	Gholaminejad et al. (2021)
	lncRNA-G21551	exosomes from the plasma	↓	significantly and differentially expresses in plasma exosomal lncRNAs of IgAN patients and their first-degree relatives	acts as a non-invasive biomarker for IgAN	Guo et al. (2020)
MN	Neat1	MN renal tubule tissues	↑	activates the Bcl-2 homology 3-only (BH3-only) protein	increases renal tubular epithelial cell apoptosis and decreased proliferation	Pi et al. (2021)
	XIST	kidney tissues of patients with membranous nephropathy	↑	regulates miR-217-TLR4 pathway	promotes podocyte apoptosis and the development of MN	Jin et al. (2019)
		kidney tissues, ascites urine, podocytes	↑	a reduction of H3K27me3 at XIST promoter regions leads to elevated levels of urinary XIST	acts as a potential biomarker for membranous nephropathy	Huang et al. (2014)
LN	lincRNA-p21	LN mononuclear and urine cells	↑	contributes to apoptotic cell death in circulating lymphocytes and renal cells	followed by production of autoantibodies, resulting in in situ IC accumulation and the formation of GN in SLE	Chen et al. (2020)
	LncRNA RP11-2B6.2	kidney tissues in mouse	↑	enhances IFN stimulated genes (ISGs) expression and epigenetically inhibits the expression of gene SOCS1	positively regulates IFN-I pathway and correlated with disease activity and IFN scores	Liao et al. (2019)
	NEAT1	human renal mesangial cells (HRMCs)	↑	modulates the miR-146b/TRAF6/NF-κB axis	accelerates renal mesangial cell injury	Zhang et al. (2020)
	TUG1	kidney of SLE mouse	↓	inhibits the apoptosis and the release of inflammatory factors, related to NF-κB inhibition	protects the HK-2 cell from LPS-induced inflammatory injury	Cao et al. (2020a)
	Lnc-FOSB-1:1	neutrophils of SLE patients	↓	indicates higher risk of future renal involvement	acts as a potential biomarker for prediction of near future renal involvement	Cai et al. (2021)
	Linc0597	serum in a LN patients	↑	unknown	be used as the indicators for disease activity and diagnosis of LN	Zheng et al. (2020)
	Lnc-DC	plasma	↑	unknown	discriminates LN from SLE without nephritis	Wu et al. (2017)
DN	CES1P1	DM model in C57BL/6 mice	↑	decreases expression of miR-214-3p, increases expression of the inflammatory factors IL-17, NF-κB, and IL-6	leads to the development of DN	Zhang et al. (2022)
	MEG3-205	db/db mice, DN patients, and AGEs-treated mesangial cells	↑	acts as a competing endogenous RNA by binding with let-7a and thus regulate MyD88	promotes renal inflammation and fibrosis	Luo et al. (2022)
	TUG1	conditionally immortalized mouse podocyte clonal cells treated with high glucose	↑	sponges miR-9 and upregulates SIRT1	protects podocytes from high glucose-induced apoptosis and mitochondrial dysfunction	Lei et al. (2022)
	NEAT1	SV40 MES13 mouse mesangial cell	↑	targets miR-124 and Capn1/β-catenin signaling pathways	increases mouse mesangial cell viability, inflammation and fibrosis	Zhao et al. (2022)
		V40 mesangial cells (MES)13 cells exposed to high concentration of glucose	↑	modulates miR-423-5p/GLIPR2 axis	accelerates the proliferation, oxidative stress, inflammation and fibrosis and suppresses the apoptosis	Wu et al. (2022)
	NEAT2	HK-2 cells treated with high glucose	↑	regulates miR-206	modulates pyroptosis of renal tubular cells induced by high glucose	El-Lateef et ak. (2022)
	MALAT1	HK-2 cells treated with high glucose	↑	activates the AMPK/mTOR signaling via interacting with LIN28A to stabilize Nox4 mRNA	aggravates high glucose-induced renal tubular epithelial injury	Song et al. (2022)
	MIAT	patients with DN	↑	increases Sox4 expression and regulated p53 ubiquitination and acetylation, thereby inhibiting the downstream factors cyclinB/cdc2 by enhancing p21cip1/waf1 activity	enhances glomerular podocyte injury and mitotic dysfunction, eventually exacerbating proteinuria	Wang et al. (2022b)
				interacts with Sox4 by sponging miR-130b-3p		
	HOTAIR	high glucose-induced human mesangial cells	↑	regulates miR-147a/WNT2B axis	facilitates high glucose-induced mesangial cell proliferation, fibrosis and oxidative stress	Wang et al. (2022a)
	UCA1	DN rat model	↓	targets miR-206	inhibits renal tubular epithelial cell apoptosis	Yu et al. (2022)
	ZFAS1	human glomerular mesangial cells induced with high glucose	↑	regulates miR-588/ROCK1 axis	regulates the proliferation, oxidative stress, fibrosis, and inflammation	Geng et al. (2022)
AKI	CASC2	serum of patients with sepsis	↓	inversely correlated with pro-inflammatory miR-155 and regulates NF-κB pathway	as a potential target for treating sepsis‑induced AKI	Wang et al. (2020a)
		sepsis cell model	↓	regulates miR-545-3p/PPARA Axis	CASC2 overexpression ameliorates sepsis-associated AKI	Hu et al. (2021)
	TapSAKI	urine-derived sepsis rat model	↑	regulates miR-22 /PTEN/TLR4/NF-κB pathway	promotes apoptosis and inflammation of HK-2 cells	Shen et al. (2019)
	HOXA-AS2	patients with sepsis and experimental models	↓	targets miR-106b-5p and hinders the Wnt/β-catenin and NF-κB pathways	protects HK-2 cells from lipopolysaccharide (LPS) -induced damage	Wu et al. (2020)
	MEG3	LPS-induced AKI animal and cell models	↑	regulates miR-18a-3p/GSDMD pathway	promotes renal tubular epithelial cell pyroptosis	Deng et al. (2021)
	DLX6-AS1	serum of patients with sepsis	↑	regulates miR-223-3p/NLRP3 pathway	promotes the cytotoxicity and pyroptosis of HK -2 cells	Tan et al. (2020)
	MALAT1	LPS-induced AKI model	↑	acts as an RNA sponge for miRNA-135b-5p to positively regulate NLRP3	participates in HK-2 cell pyroptosis and inflammation	Huang and Xu (2021)
	GAS5	sepsis-induced AKI model	↓	inhibits miR-579-3p, activates SIRT1/PGC-1α/Nrf2 signaling pathway	participates in AKI-related pyroptosis	Ling et al. (2021)

LncRNA affects DN through EMT, endothelial-to-mesenchymal transition (EndMT), and actions on miRNAs. MALAT1, NR_033515, Erbb4-IR, GAS5, and CJ241444 are involved in tubular injury and contribute to EMT, while MIAT and LncRIAN show tubular protective activity and may regulate EMT in diabetic kidneys. MALAT1, Erbb4-IR, and ASNCMTRNA2 cause EC injury and may be involved in EndMT-related renal fibrosis. The lncRNA H19 is associated with renal fibrosis by activating the EndMT process in diabetes (Xia W. et al., 2021; Srivastava et al., 2021). Some lncRNAs also act as miRNA sponges by preventing miRNA binding to affect miRNA function by binding to the actual target mRNA. Both EndMT and miRNA are affected by oxidative stress, again suggesting that oxidative stress affects many DN processes (Giordo et al., 2021). In DN mice, lncCLYBL-AS2 inhibited by Coptis improved EMT and fibrogenesis in HK-2 cells through the miR-204-5p/SNAI1 axis (Cai et al., 2020). Jixuepaidu Tang-1 inhibits EMT and alleviates renal damage by suppressing the lncRNA LOC498759 (Jin J. et al., 2019).

**FIGURE 3:**
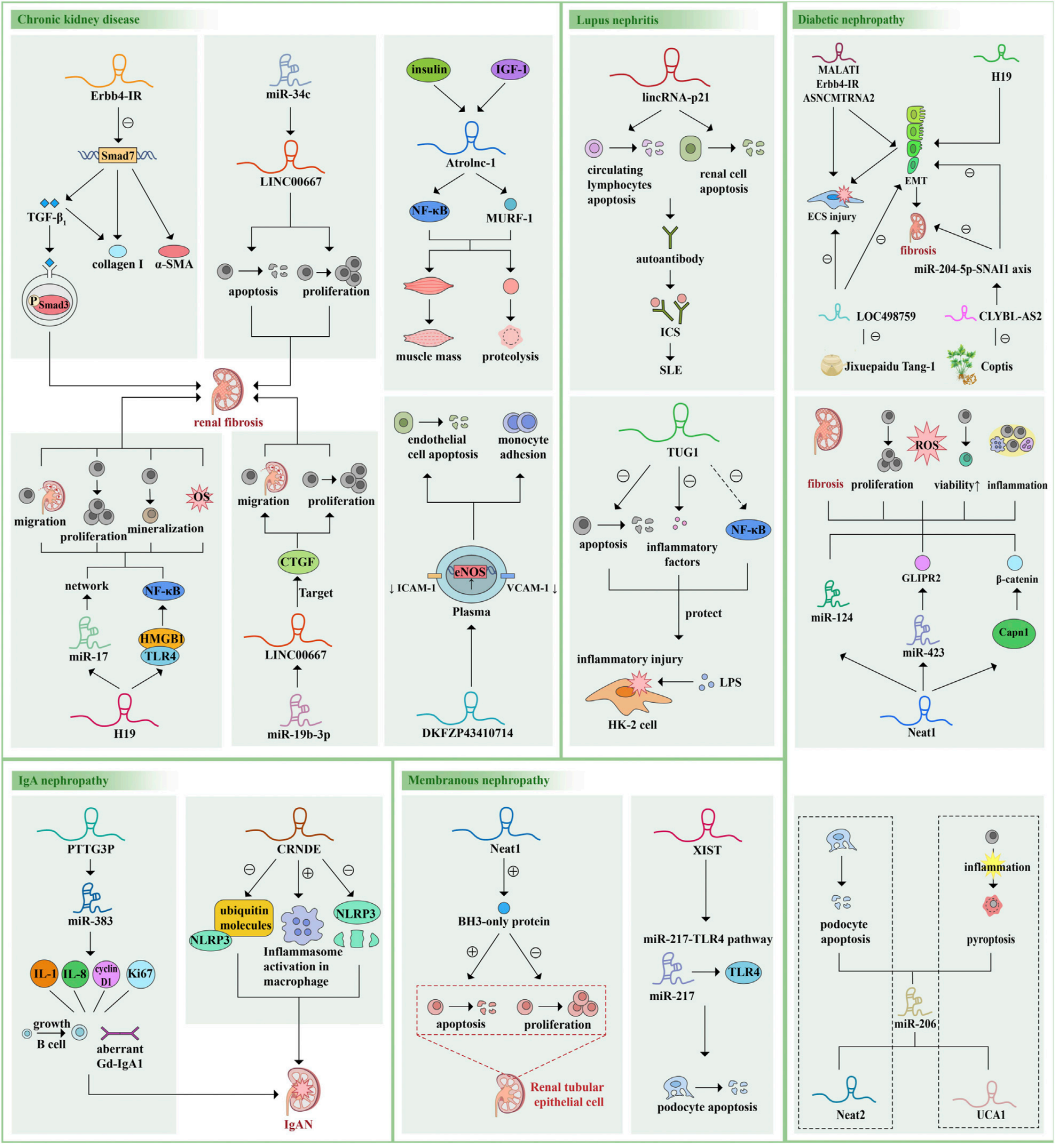
Examples of the roles of lncRNAs in kidney disease pathophysiology. LncRNAs are mainly involved in the occurrence and development of various kidney diseases by regulating downstream miRNAs or genes, regulating different pathways and signal transduction axis, sponging miRNAs and changing protein molecules and cytokine levels, thus regulating cell proliferation, apoptosis, pyroptosis, migration, oxidative stress, EMC and changing cell vitality. Here we give some more studied examples of lncRNAs and their mechanisms of action and functions. The same lncRNA can be involved in different diseases and act on different pathways or miRNAs. Different lncRNAs can also act on the same downstream target, forming a vast regulatory network. α-SMA, alpha-smooth muscle actin; TGF-β, transforming growth factor-β; IGF-1, insulin like growth factor-1; NF-κB, nuclear factor kappa B; MURF-1, muscle ring finger 1; HMGB1, high mobility group box 1; TLR4, Toll-like receptor 4; CTGF, connective tissue growth factor; ICAM-1, intracellular adhesion molecule 1; VCAM-1, vascular cells adhesion molecule 1; eNOS, endothelial nitric oxide synthase; ICs, immune complexes; SLE, systemic lupus erythematosus; LPS, lipopolysaccharide; IL, interleukin; Gd-lgA1, galactose-deficient IgA1; GLIPR2, Glioma pathogenesis related-2.

We noticed that HOTAIR is expressed in normal adult glomerular podocytes and was also found to be up-regulated in the kidneys of DM patients and mice; however, knockdown of HOTAIR from podocytes has little effect on glomerular injury in diabetic mice. The gene of this lncRNA is similar to the adjacent gene, suggesting that the structure of lncRNA is very similar, and the lncRNA with a changed expression level may be a bystander rather than a facilitator of the disease process, so we should be cautious of the relationships between lncRNAs and disease (Majumder et al., 2019; Ren and Wang, 2021; Chu et al., 2022) (Figure 3).

## LncRNA and AKI

LncRNA regulates cell viability, the secretion of cytokines or inflammatory factors in AKI, apoptosis, pyroptosis, and oxidative stress by targeting miRNA and acting on corresponding pathways and participates in the pathophysiological process of AKI.

Sepsis caused by infection often leads to systemic multiorgan damage, including sepsis-associated AKI, and AKI has a high probability of progressing to CKD and ESKD. Susceptibility cancer candidate 2 (CASC2) expression was significantly reduced in sepsis patient serum and LPS-treated HK2 and HEK293 cells, with levels inversely correlating with the severity of AKI and pro-inflammatory miR-155. CASC2 has been shown to promote cell viability and inhibit secretion of inflammatory factors, apoptosis, and oxidative stress in LPS-stimulated human renal tubular epithelial HK-2 cells (Hu et al., 2021). CASC2 may serve as a potential target for the treatment of sepsis-induced AKI by inhibiting miR-155-and NF-κB pathway–mediated inflammation (Wang M. et al., 2020). CASC2 overexpression ameliorates sepsis-associated AKI by regulating the miR-545-3p/PPARA axis (Hu et al., 2021). It was also observed that the lncRNA TapSAKI promotes the expression of phosphatase and tensin homolog (PTEN), TLR4/NF-κB pathway–related proteins TLR4 and P-p65, and apoptotic protein cleaved caspase-3 through negative regulation of miR-22 in renal injury induced by urinary sepsis. The miR-22/PTEN/TLR4/NF-κB pathway promotes the inflammatory injury of HK-2 cells and myeloid PTEN deficiency aggravates renal inflammation and fibrosis (Shen et al., 2019; An et al., 2022 ). Another lncRNA, HOXA cluster antisense RNA 2, showed a protective effect in sepsis-induced AKI by targeting miR-106B-5p and blocking the Wnt/β-catenin and NF-κB pathways (Wu et al., 2020). Maternally expressed gene three controls gasdermin D expression by acting as a ceRNA of miR-18a-3p to promote tubular epithelial cell pyroptosis (Deng J. et al., 2021). Other lncRNAs, such as lncRNA DLX6 antisense RNA 1, MALAT1, PVT1, and GAS5, are involved in pyroptosis in sepsis-associated renal injury (Tan et al., 2020; Deng L. T. et al., 2021; Huang and Xu, 2021; Ling et al., 2021). Resveratrol rescues the viability and cytokine release of LPS-treated HK2 cells by inactivating the lncRNA MALAT1/miR-205 axis and attenuates sepsis-induced AKI (Wang B. et al., 2021).

In addition to sepsis, ischemia/reperfusion (IR) is another cause of AKI. NEAT1 is highly expressed in IR-induced AKI, which can induce apoptosis of renal tubular epithelial cells by down-regulating miR-27a-3p and enhancing ischemia-induced injury (Jiang et al., 2019). The lncRNAs H19 and TUG1 show bidirectional regulation of cellular functions in IR-induced AKI (Yang et al., 2022). A recent study investigated whether total glucosides of paeony inhibited autophagy and improved AKI induced by IR *via* the TUG1/miR-29a/PTEN axis (Chang et al., 2021).

Kidney transplantation and toxicity of cisplatin and other drugs can also cause AKI. Previous research has established that lncRNAs are involved in the process of AKI, and some have renal protective effects and bidirectional regulation of cell function (Zhou Q. et al., 2019; Ignarski et al., 2019; Ren et al., 2019; Yang et al., 2022).

## LncRNAs in complications of kidney diseases

### Muscle atrophy

Chronic renal disease stimulates skeletal muscle protein degradation pathways while activating mechanisms that impair muscle protein synthesis and repair. Loss of muscle proteins is a deleterious consequence of kidney diseases, which leads to a decrease in muscle strength and function and may reduce the quality of life, for example by causing cachexia. Signaling molecules secreted by muscle can enter the circulation and subsequently interact with receptor organs, including the kidneys. Conversely, pathological events in the kidneys can adversely affect protein metabolism in skeletal muscle, demonstrating a reciprocal communication between kidneys and muscle (Wang et al., 2022). The process is complex and caused by impaired IGF-1/insulin signaling, metabolic stress, elevated glucocorticoids, and inflammation (Sun et al., 2018). An increase in atrophy-related lncRNA-1 (Atrolnc-1) was found in the muscles of mice with CKD. The overexpression of Atrolnc-1 up-regulated the expression of genes related to atrophy, while its depletion prevented CKD-induced muscle mass loss and proteolysis (Liu et al., 2021). The A20 binding inhibitor of NF-κB-1 is a protein that inhibits NF-κB signaling. Atrolnc-1 interacts with this binding inhibitor and blocks its inhibitory ability, leading to the activation of NF-κB signaling. NF-κB, a key transcription factor in the inflammatory response (Verzola et al., 2017), continuously promotes the expression of ubiquitin E3 ligase muscle ring finger 1 through the ubiquitin–proteasome system to increase muscle proteolysis (Sun et al., 2018).

### CVD

Cardiovascular events are the main cause of death in patients with ESRD, and the incidence of CVD in patients with CKD is 20–30 times greater than that in patients without CKD (Schiffrin et al., 2007). An essential pathological manifestation of CKD is vascular calcification. LncRNAs may alleviate arterial calcification through certain mechanisms and also play protective roles (Wang et al., 2016; Shi et al., 2018; LincRNA-p21, 2019; Zhang X. et al., 2020).

LncRNAs can reprogram cells by switching gene-expression patterns. H19, one of the most frequently studied lncRNAs, is up-regulated in patients with intermediate and advanced CKD and in calcified aortic valve disease. NOTCH1 controls osteogenic activity in the aortic valve and regulates the expression of key pro-osteogenic genes such as *RUNX2* and *BMP2*. Gene-promoter analysis revealed that H19 silences NOTCH1 by blocking the recruitment of p53 to the promoter of NOTCH1. Knockdown of H19 in interstitial valve cells increases the expression of NOTCH1 and subsequently decreased the levels of its repressed downstream targets, *RUNX2* and *BMP2*, thereby delaying aortic valve osteogenesis (Hadji et al., 2016). Overexpression of ANCR, another lncRNA, significantly down-regulates the expression of RunX2 and BMP-2 in β-glycerophosphate–stimulated vascular smooth muscle cells (VSMCs), promoting the expression of autophagy-related markers LC3 and Atg5, and inhibits the osteogenic differentiation of VSMCs. ANCR may inhibit the osteogenic differentiation of VSMCs by activating autophagy, thereby attenuating arterial calcification (Cao L. et al., 2020). The lncRNA GAS5 is significantly down-regulated in calcified human aortic VSMCs. GAS5 may bind to miR-26-5p, and PTEN is a direct target of miR-26b-5p. Overexpression of GAS5 significantly attenuates osteogenic differentiation and calcification of human aortic VSMCs induced by high phosphorous levels. This suggests that the GAS5/miR-26-5p/PTEN axis can serve as a potential therapeutic target for vascular calcification in patients with CKD (Chang et al., 2020).

EC damage and toxin accumulation in kidney disease also induce CVD. In the blood of DM/CKD patients, endothelial damage and the accumulation of uremic toxins are known mechanisms of CVD pathogenesis. In a study of DM/CKD-induced CVD, it was found that, after indoxyl sulfate treatment, SLC15A1-1 expression was up-regulated, which accelerated pro-inflammatory macrophage activation and further induced vascular inflammation in CKD. This novel pathway may be responsible for the endothelial inflammation that leads to increased CVD in DM/CKD and may be a therapeutic target for future clinical applications (Huang Y. C. et al., 2021).

There is a communication process of exosomal lncRNAs between CKD and CVD, and the exosome-derived lncRNA ZFAS1 controls cardiac fibrosis in CKD. Studies have shown increased expression of ZFAS1 in the hearts of CKD mice, and transfection of ZFAS1 into human cardiomyocytes (HCMs) and the collection of exosomes revealed significant enrichment of ZFAS1 in HCM-derived exosomes. Injection of HCM-derived exosomes into mice significantly reduced their cardiac function and induced myocardial fibrosis. Biological databases and luciferase assays suggest that ZFAS1 is transferred to human cardiac fibroblasts *via* HCM-secreted exosomes and induces myocardial fibrosis *via* the miR-4711-5p/WNT/β signaling pathway (Wang Y. et al., 2021).

LncRNA is also differentially expressed in different disease combinations and complications and could be a predictive marker for cardiovascular complications in kidney disease. Long intergenic ncRNA predicting cardiac remodeling (LIPCAR) has emerged as a promising lncRNA biomarker for cardiac disease and remodeling. Circulating LIPCAR is increased in heart failure patients with impaired renal function (Meessen et al., 2021). The analysis of lncRNAs in plasma from ESRD or CKD patients and healthy subjects revealed that plasma lncRNA-expression profiles were able to distinguish ESRD from CKD and control samples. Analysis of microarray datasets obtained from kidney biopsy samples from patients with advanced kidney disease showed that elevated plasma lncRNA DKFZP434I0714 levels were a significant independent predictor of adverse cardiovascular outcomes in uremic patients. This suggests that lncRNA can predict adverse cardiovascular outcomes in patients with ESRD (Lai et al., 2019).

## Conclusion and perspectives

During the progression of renal disease, there will be renal function impairment; loss of nephrons; and renal fibrosis, which is characterized by the excessive accumulation of ECM components, decreased GFR, and abnormal proteinuria, eventually leading to uremia. There is no better treatment for uremia than alternative therapy, but a limited number of patients can afford such treatment. At present, lncRNAs have been identified as key molecules involved in the pathology, physiology, and pathophysiology of various renal diseases. LncRNAs are related to multiple pathways of renal fibrosis, the most classical of which is the TGF-β/Smad pathway, it is also associated with oxidative and inflammatory damage in renal fibrosis, including ROS and inflammasomes, and regulates apoptosis, proliferation, and pyroptosis of renal cells. Numerous studies have found that lncRNAs are differentially expressed, and research on lncRNA is moving toward precision medicine.

New biomarkers are urgently needed in the field of kidney disease to achieve better prevention and prognosis, but current studies have limitations. First, few therapeutic drug studies targeting lncRNAs are available; thereby, further studies are needed to determine the role of lncRNAs as molecular drug targets in animal models and to validate promising biomarker candidates in multicenter trials. In addition, lncRNAs are involved in the occurrence and development of abundant kidney disease events, but many mechanisms are unclear and puzzles remain to be solved by researchers. LncRNAs can regulate disease processes through epigenetic regulation, but the mechanisms at play are unknown, and lncRNAs also have an intimate connection with genetic factors, consistent with their regulation at the epigenetic level, which may be another direction for the prevention and treatment of hereditary nephropathy. In the ceRNA network, lncRNAs are able to interact with multiple targets at the same time, thus affecting a large number of cellular pathways and producing a cascade effect, and the structures of lnRNAs are very similar and different lncRNAs may target unified targets (e.g., UCA1 and NEAT2 both target miR-206); thus, there is no guarantee of a 1-to-1 correspondence between an lncRNA and a mechanism or disease, and changes in the expression level of an lncRNA do not necessarily indicate its involvement in this event, which should be treated with caution during the course of the study. It is worth noting that most studies are still in a stage of theoretical research or animal experimentation; there persists a gap between the theory and application of lncRNAs as interventions in kidney diseases, which researchers must cross.

## Glossary

ESRD: end-stage renal disease
LncRNA: Long non-coding RNA
CKD: chronic kidney disease
MN: membranous nephropathy
IgA: Immunoglobulin A
IgAN: immunoglobulin A nephropathy
LN: Lupus nephritis
DN: diabetic nephropathy
AKI: acute kidney injury
Pol II: polymerase II
lincRNAs: long intergenic non-coding RNAs
snoRNA: small nucleolar RNA
rDNA: ribosomal DNA
RBP: RNA-binding protein
ceRNA: competing endogenous RNA
UUO: unilateral ureteral obstruction
TGF-β: transforming growth factor-β
TSI: TGF-β/Smad3 interacting long non-coding RNA
PVT1: plasmacytoma variant translocation 1
IL: interleukin
NF-κB: nuclear factor kappa B
Erbb4-IR: Erbb4-immunoreactivity
XIST: X inactive specific transcript
TLR4: Toll-like receptor 4
TNF-α: tumor necrosis factor-α
SOX6: SRY-Box Transcription Factor 6
PTEN: phosphatase and tensin homolog
MIAT: myocardial infarction-associated transcript
EMT: epithelial-mesenchymal transition
HOTAIR: homeobox transcript antisense RNA
RIF: renal-interstitial fibrosis
caspase-1: aspartate-specific proteases-1
MALAT1: metastasis-associated lung adenocarcinoma transcript 1
GAS5: growth arrest-specific 5
F2: filial generation 2
CRNDE: colorectal neoplasia differentially expressed
NLRP3: NOD-like receptor protein 3
NOD: nucleotide-binding oligomerization domain
H3K27me3: trimethylation of histone H3 at lysine 27
miR/miRNA: microRNAs
SOX4: Box transcription factor 4
SLE: systemic lupus erythematosus
ICs: immune complexes
IFN-I: type I interferon
TUG1: taurine up-regulate gene 1
LPS: lipopolysaccharide
DM: diabetes mellitus
ECS: endothelial cell
EndMT: endothelial-to-mesenchymal transitions
CASC2: cancer candidate 2
HOxA-AS2: HOXA cluster antisense RNA 2
IR: ischemia/reperfusion
IGF-1: insulin like growth factor-1
Atrolnc-1: Atrophy-related long non-coding RNA-1
MURF-1
muscle ring finger 1
CVD: cardiovascular disease
VC: vascular calcification
VSMC: vascular smooth muscle cell
HCM: human cardiomyocytes
LIPCAR: Long Intergenic non-coding RNA Predicting cardiac remodeling
